# Rapid evolution of pyrethroid resistance prevalence in *Anopheles gambiae* populations from the cities of Douala and Yaoundé (Cameroon)

**DOI:** 10.1186/s12936-015-0675-6

**Published:** 2015-04-14

**Authors:** Christophe Antonio-Nkondjio, Billy Tene Fossog, Edmond Kopya, Yacouba Poumachu, Benjamin Menze Djantio, Cyrille Ndo, Timoléon Tchuinkam, Parfait Awono-Ambene, Charles S Wondji

**Affiliations:** Laboratoire de Recherche sur le Paludisme, Organisation de Coordination pour la lutte Contre les Endémies en Afrique Centrale (OCEAC), PO Box 288, Yaoundé, Cameroon; Faculty of Sciences, University of Yaoundé I, PO Box 337, Yaoundé, Cameroon; Vector Group, Liverpool School of Tropical Medicine, Pembroke Place, Liverpool, L3 5QA UK; Malaria Research Unit of the Laboratory of Applied Biology and Ecology (MRU-LABEA), Department of Animal Biology, Faculty of Science of the University of Dschang, PO Box 067, Dschang, Cameroon; Faculty of Medicine and Pharmaceutical Sciences, University of Douala, Douala, Cameroon

**Keywords:** *Anopheles gambiae s.l*, pyrethroid resistance, malaria, intron-1, Yaoundé, Douala, Cameroon

## Abstract

**Background:**

The adaptation of malaria vectors to urban areas is becoming a serious challenge for malaria control. The study presents the evolution of pyrethroid resistance in mosquito populations from the cities of Douala and Yaoundé between 2010 and 2013.

**Methods:**

Susceptibility tests to permethrin and deltamethrin were carried out with two- to four-day old unfed *Anopheles gambiae sensu lato* adults raised from larvae collected from the field. Mosquitoes resistant to permethrin and deltamethrin and control were screened to detect the presence of the *kdr* alleles using the TaqMan assays. Mosquitoes belonging to the *An. gambiae* complex were subjected to PCR assays designed for species and molecular forms identifications. The genomic region containing the upstream of intron-1 of the voltage-gated sodium channel was sequenced and compared between mosquitoes originating from different breeding habitats.

**Results:**

*Anopheles gambiae s.l.* specimens collected from the city of Douala were all *Anopheles coluzzii*. In Yaoundé, both *An. gambiae* and *An. coluzzii* were recorded. A rapid decrease of mosquito mortality to permethrin and deltamethrin was recorded between 2010 and 2013 in the two cities. The mortality rate varied from 80.3 to 22.3% and 94.4 to 59.7% for permethrin and deltamethrin, respectively. Both *kdr* alleles L1014F and L1014S were recorded. The frequency of *kdr* alleles increased rapidly over the study period, varying from 44 to 88.9% in Yaoundé and from 68 to 81% in Douala. The sequencing of a 1,228 bp region of intro-1 of the voltage-gated sodium channel revealed the presence of five different haplotypes. A high number of these haplotypes were recorded in *An. coluzzii* samples. No evidence for a recent selective sweep on intron-1 sequence within samples originating from different breeding habitat was detected using Fu’s and Tajima Fs statistics.

**Conclusion:**

The present study supports rapid evolution of pyrethroid resistance in vector populations from the cities of Douala and Yaoundé and calls for immediate action to fight against the increasing prevalence of pyrethroid-resistant mosquitoes.

## Background

In the absence of vaccine, malaria prevention relies almost exclusively on vector control and pyrethroids are the most commonly used insecticides for vector control [1]. Their rapid knockdown effect, high remanence and low toxicity for humans at operational doses make them ideal compounds for both bed-net impregnation and indoor residual spraying (IRS). Pyrethroids have as target the insect nervous system and act by inducing a long-term disruption of the sodium permeability which alters the properties of the sodium channel and causes the insect death [2]. However, rapid evolution of pyrethroid resistance resulting from increased use of these compounds in both public health and agriculture is affecting the efficiency of control measures [3]. During the past decades, the use of pyrethroid-based interventions, IRS and/or long-lasting insecticidal nets (LLINs), dramatically increased across the continent and contributed in the reduction of malaria morbidity and mortality [4,5]. There is concern that the expansion of pyrethroid resistance could jeopardize current momentum of reduction and elimination of malaria [6]. Two main physiological mechanisms confer pyrethroid resistance, these are: target site insensitivity and metabolic resistance [7]. However target site resistance remains the most studied because of easier means of assessment [8-10]. Mutations of *kdr* gene, both leucine to phenylalanine substitution (L1014F) and leucine to serine substitution (L1014S) are the two mutations inducing target site resistance in insects [11,12]. These two mutations are now largely distributed across the continent, yet differences in the prevalence of these resistance alleles have been reported between *Anopheles gambiae s.l.* species and between sites [13-15]. The genotyping of the *kdr* locus and partial sequencing of the upstream intron-1 of the voltage-gated sodium channel gene of mosquitoes collected across sub-Saharan Africa also provided evidence for the high genetic diversity of this gene [13]. Although the importance of *kdr* mutations as a stand-alone mechanism conferring pyrethroid resistance is still subject to debate [16], the current evolution of pyrethroid resistance and the diversity of resistance mechanisms represent a great challenge that requires further attention.

In Cameroon, despite the introduction of pyrethroid-treated nets (ITNs) in the late 1990s, a fast evolution of resistance was recorded across the country a few years after implementation [17,18]. The increased use of pesticides in agriculture, the expansion of urban agriculture and pollution in urban settings, were also reported to influence the prevalence and distribution of insecticide resistance across the country [19,20]. In the cities of Douala and Yaoundé, *An. gambiae* larvae emerging from cultivated and organically polluted sites were found to be 325 and 193 times, respectively, more tolerant to deltamethrin than *An. gambiae* Kisumu larvae [21]. Despite the fact that pyrethroid resistance is dynamic, the influence of local selective pressure induced either by agricultural practices, urban pollution or even increase use of ITNs on the evolution of resistant alleles remain poorly understood. Analysis conducted with mosquitoes originating from different type of habitats in the city of Yaoundé suggested a different resistance pattern in mosquito populations according to their breeding habitat origins [22]. The present study assessed the evolution of insecticide resistance from 2010 to 2013 in the cities of Douala and Yaoundé and the variability of intron-1 of the voltage-gated sodium channel gene in mosquitoes originating from different larval habitats.

## Methods

### Study sites

The study took place in the cities of Yaoundé (3°51’N11°30’E) and Douala (3°48’N10°08’E), the two major urban cities in Cameroon. Douala and Yaoundé have a population approaching three million inhabitants each [23]. These cities are situated within the Congo-Guinean phytogeographic zone characterized by a typical equatorial climate, with two rainy seasons extending from March to June and from September to November. Douala is situated near the Atlantic coast 1 m above sea level and receives over 3,500 mm of rainfall annually, whereas the annual average rainfall in Yaoundé is 1,700 mm. Yaoundé is located inland 250 km east of Douala. The city is situated 800 m above sea level and is surrounded by many hills. The study was conducted under the ethical clearance N° 216/CNE/SE/09 delivered by the Cameroon National Ethics Committee Ref N° IORG0006538-IRB00007847-FWA00016054.

### Insecticide susceptibility assays

Bioassays were carried out using WHO test kits for adult mosquitoes [24]. The following diagnostic concentrations of insecticides were tested: 0.75% permethrin and 0.05% deltamethrin.

Susceptibility tests were carried out with two to four days old unfed *An. gambiae s.l.* adults raised from larvae collected in breeding sites. Batches of 20 to 25 mosquitoes per tube were exposed to impregnated papers for one hour. The number of mosquito knockdown was recorded every 5 minutes during exposure. After exposure, mosquitoes were supplied with glucose solution as food, and mortality was recorded 24 hours post-exposure. Tests with untreated papers were systematically run as controls. Mortality rate in tested samples was corrected using Abbot formula [25] when the mortality rate of control was between 5 and 20%. WHO criteria [24,26] were used to evaluate the resistance and susceptibility status of the tested mosquito population. The resistance status was indicated by a mortality rate below 90% while mortality rates greater than 97% were indicative of susceptibility, and mortality rates between 90 and 97% suggested increased tolerance, but resistance should be confirmed.

To screen for the presence of the *kdr* alleles (L1014F and L1014S) conferring resistance to DDT and pyrethroids, DNA extracted from individuals exposed to insecticide was tested using the hot ligation oligonucleotide assay (HOLA) of Lynd *et al.* [8] and/or the TaqMan assay [26].

### Mosquito identification

Anopheline larvae were identified morphologically using the Gillies and Coetzee keys [27]. Mosquitoes belonging to the *An. gambiae* complex were subjected to PCR assays designed for species and molecular forms identifications [28]. Genomic DNA used for molecular analysis was extracted from dessicated adult mosquitoes according to either Livak [29] or Cornel [30] protocols.

### Intron sequence determination

Mosquito used for sequencing included specimen originating from organically polluted, unpolluted and agricultural cultivated sites. The genomic region containing the upstream of intron-1 was PCR amplified and sequenced as presented in Weill *et al.* [31]. PCR products were then purified using the QIAquick purification Kit (Qiagen, Hilden, Germany). The sample was afterward sequenced on both strands. Sequences from each specimen were analysed manually to detect polymorphic sites using the software BIOEDIT V: 7.0.5.2. Sequence differences in multiple alignments were analysed using Clustal O.

### Data analysis

The software DnaSP 5.10 [32] was used to estimate the genetic polymorphism of intron-1 in samples originating from different breeding habitats. Parameters measured were respectively: the number of haplotypes, haplotype diversity, the number of segregating sites, Fu’s Fs statistics, Tajima statistics.

For bioassay analyses, comparison of percentages was performed using the Chi square test. Estimates of mortality rates and the 95% confidence interval were determined using the software MedCalc V11.5.0.0.

## Results

### Evolution of *Anopheles gambiae s.l.* susceptibility to permethrin and deltamethrin in different sites across Cameroon

**Figure 1:**
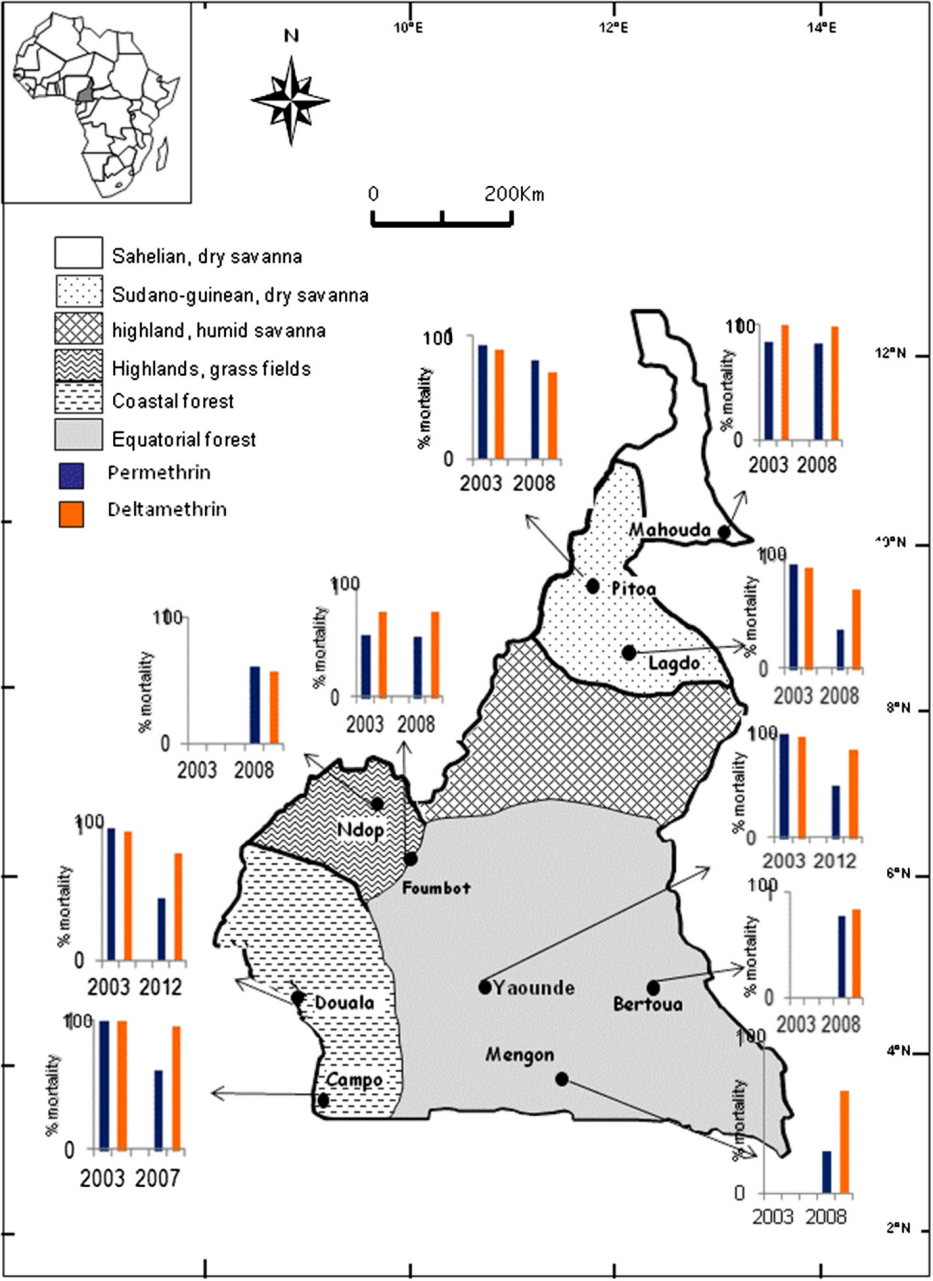
A map showing the evolution of *Anopheles gambiae* susceptibility to permethrin and deltamethrin between 2003 and 2012 in different parts of Cameroon.

A map describing the evolution of *An. gambiae s.l.* susceptibility to permethrin and deltamethrin from 2003 onwards after the first countrywide free distribution of ITNs is presented (Figure 1). Data presented here have been extracted from the present study and from different published reports [17,19,33-35]. For a better understanding of the pattern of *An. gambiae s.l.* susceptibility it is important to keep in mind that, the northern half of the country situated in dry savanna and humid savanna areas have as dominant malaria vector *Anopheles arabiensis* whereas, *An. gambiae* and *An. coluzzii* are the main vectors in the southern half of the country (highlands, coastal and equatorial forest regions).

### Annual evolution of *Anopheles gambiae* susceptibility to pyrethroids in Yaoundé and Douala between 2010 and 2013

**Figure 2:**
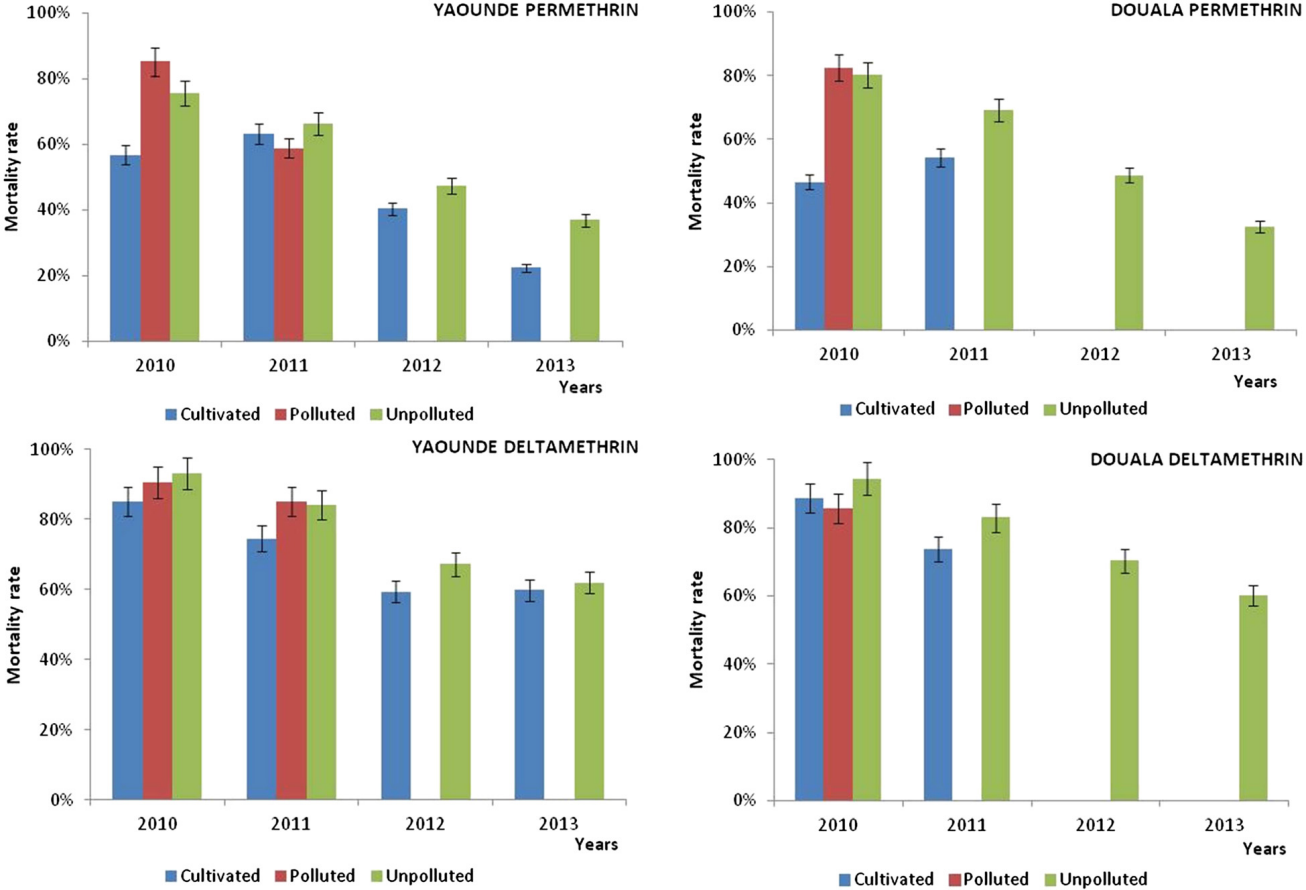
Evolution of *Anopheles gambiae s.l.* susceptibility to permethrin and deltamethrin from 2010 to 2013 in the cities of Douala and Yaoundé.

To assess the evolution of mosquito susceptibility to permethrin and deltamethrin, susceptibility assays were conducted at least twice each year at different seasons from 2010 to 2013. Data from 2010 have already been published [35], but are included here for comparison. Apart of mosquitoes collected in cultivated sites, mosquitoes collected in either polluted or non-polluted sites in both Douala and Yaoundé, showed high susceptibility to permethrin at the beginning of the study with a mortality rate ranging from 75 to 85% in 2010. Four years after, less than 40% of *An. gambiae s.l.* specimens in both cities were detected still susceptible to permethrin, this showing the total establishment of permethrin resistance in these species populations (Figure 2). Deltamethrin mortality decreased significantly between the beginning and the end of the study with mortality rates varying from 95% in 2010 to about 60% in 2013 in both cities (Figure 2). When quantifying the decrease in vector susceptibility between 2010 and 2013, it appeared that *An. gambiae s.l.* was four to eight times and three to eleven times less susceptible to permethrin and deltamethrin, respectively, in 2013 compared to 2010 (Table 1).

**Table 1:** **Comparison of the level susceptibility of mosquitoes to permethrin and deltamethrin between 2010 and 2013 in Yaoundé and Douala**

**Insecticide**	**City**	**Site**	**2010**	**2013**	**Odds ratio (2010** ***vs*** **2013)**	**P value**
						**(2010** ***vs*** **2013)**
			**% mortality**	**% mortality**		
			**(95%** **CI)**	**(95%** **CI)**		
Permethrin	Douala	Unpolluted	80.3% (73–88.2)	32.5% (28–37.5)	8.47	<0.00001
	Yaoundé	Unpolluted	75.6% (66.7-85.3)	36.8% (48.8-61.1)	5.31	<0.00001
	Yaoundé	Cultivated	56.7% (43.2-73.2)	22.3% (14.2-33.5)	4.56	<0.00001
Deltamethrin	Douala	Unpolluted	94.4% (87.5-100)	60% (54.1-66.4)	11.35	<0.00001
	Yaoundé	Unpolluted	93.1% (82.8-100)	61.9% (55.1-69.4)	8.26	<0.00001
	yaoundé	Cultivated	85% (71.6-100)	59.7% (48.2-73.3)	3.83	<0.00001

### Evolution of *kdr* alleles prevalence in mosquito populations

**Figure 3:**
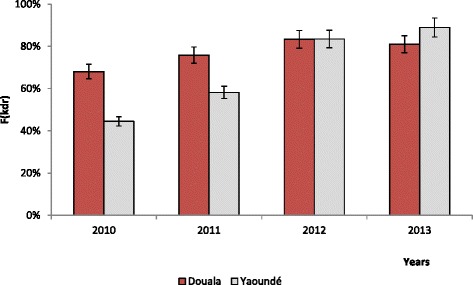
Evolution of the *kdr* allele frequency (F(*kdr*)) in *Anopheles gambiae s.l.* populations between 2010 and 2013 in Douala and Yaoundé.

Both *kdr* alleles L1014F and L1014S were recorded in Douala and Yaoundé. The frequency of *kdr* alleles increased rapidly over the four-year period in the city of Yaoundé, varying from 44% in 2010 to over 88.9% in 2013. In Douala, a similar trend was recorded with the allele frequency varying from 68 to 81% during the same period (Figure 3). Of the 1,449 mosquitoes processed during the study period, eight were found carrying the L1014S allele (seven in Douala and one in Yaoundé) and 1,200 (82.8%) were found with the L1014F allele.

### Species identification

A total of 1,688 samples were identified to species level. Of the 871 mosquitoes processed in Yaoundé, 741 (85%) were *An. coluzzii* and 130 (15%) *An. gambiae*. In Douala, of the 817 mosquitoes processed 814 (99.6%) were *An. coluzzii* and three specimens were *An. gambiae*. No change in mosquito composition was recorded during the study period in the two cities.

### Genotyping of intron-1 of voltage sodium channel in mosquito populations emerging from different type of breeding habitats

A total of 43 samples (2 N = 86) collected in 2011 were sequenced to assess the diversity of the intron-1 of the voltage-gated sodium channel in mosquitoes originating from different types of habitats. Due to the small sample size, samples from Douala and Yaoundé were merged and analysed as a unique population. The sample analysed included 15 specimens from unpolluted sites, nine from polluted sites and 19 from cultivated sites. Samples from unpolluted and polluted sites were all *An. coluzzii*. Samples from cultivated sites were constituted of eleven *An. coluzzii* and eight *An. gambiae*. The L1014F allele was detected in 12 of the 43 samples, the L1014S in one of the 43 samples, and 1014 L in 30 samples.

The sequencing of a 1,228 bp region of intron-1 revealed up to four polymorphic sites resulting in five different haplotypes (Table 2). Haplotypes MS1 and MS3 were found to be the most prevalent. Haplotype MS1 was found associated to both resistant alleles (L1014F and L1014S) and the susceptible allele (L1014L). Combinations of kdr alleles and intron-1 haplotypes in different breeding habitats are presented in Table 2. The highest number of haplotypes was recorded in *An. coluzzii* samples. Estimates of genetic variability in intron-1 showed a haplotype diversity varying from 0.43 for *An. gambiae* to 0.81 for *An. coluzzii* samples from unpolluted sites. No departure from neutrality was recorded using the Fs statistics after pooling mosquitoes according to their breeding habitats. The highest Fs value (−0.614) was recorded in samples originating from unpolluted breeding sites and this was not significant (P >0.15).

**Table 2:** **Variability of the intron-1 of the** ***kdr*** **gene in** ***Anopheles coluzzii*** **and** ***Anopheles gambiae*** **collected in 2011 in Douala and Yaoundé**

**Mutations positions**					**Unpolluted**		**Polluted**		**Cultivated**			
**702**	**703**	**896**	**Others**	**Haplotypes**	***An. coluzzii***		***An. coluzzii***	**%**	***An. coluzzii***		***An. gambiae***	
					**2 N = 30**	**%**	**2 N = 18**	**%**	**2 N = 22**	**%**	**2 N = 16**	**%**
T	C	C	-	MS1	16	53.3%	8	44.4%	4	18.2%	16	100%
T	C	A	-	M14	-	-	2	11.1%	2	9.09%	-	-
C	C	C	-	MS3	6	20%	6	33.3%	12	54.5%	-	-
C	C	C	169C	M15	4	13.3%	-	-	-	-	-	-
C	C	C	394G	M16	4	13.3%	2	11.1%	4	18.2%	-	-
Kdr alleles- Intron-1 haplotypes												
			MS1-L1014L		6	40%	3	33.3%	-	-	1	12.5%
			MS1-L1014F		2	13.3%	1	11.1%	2	18.2%	6	75%
			MS1-L1014S		-	-	-	-	-	-	1	12.5%
			MS3-L1014L		3	20%	3	33.3%	6	54.5%	-	-
			M14-L1014L		-	-	1	11.1%	1	9.1%	-	-
			M15-L1014L		1	6.67%	-	-	-	-	-	-
			M15-L1014F		1	6.67%	-	-	-	-	-	-
			M16-L1014L		2	13.3%	1	11.1%	2	18.2%	-	-
		Estimates of genetic variability										
				h	4		4		4		3	
				Hd ± SD	0.82 ± 0.034		0.76 ± 0.05		0.59 ± 0.085		0.43 ± 0.14	
				k	1.42		1.098		1.048		0.633	
				n	4		3		3		2	
				D	1.045		0.718		0.706		0.13	
				Fs	−0.614		0.089		1.43		0.062	

h: number of different haplotypes; Hd ± SD, haplotype diversity (±standard deviation); K: average number of pairwise nucleotide differences; n: number of mutations; D: Tajima’s test; Fs: Fu’s Fs statistic.

## Discussion

A fast evolution of pyrethroid resistance prevalence in vector populations of the cities of Douala and Yaoundé was recorded between 2010 and 2013. The situation was in accordance with ongoing reports across the country supporting rapid expansion of pyrethroid and DDT resistance in vector populations [19,20,36,37]. Because pyrethroid resistance was determined after mosquito exposition to reference diagnostic insecticide doses, it was not clear whether the growing prevalence of pyrethroid resistance confers mosquito-elevated capacities to withstand higher insecticide doses or longer exposure time. Hence, data recorded so far, which are the highest ever reported in the country, are consistent with an increased selective pressure on mosquito populations. The rapid drop in mosquito susceptibility to both permethrin and deltamethrin from 2012 suggests the influence of selection induced by the latest massive distribution of ITNs to the population. Since 2003, two large-scale, free distribution campaigns of ITNs were conducted in Cameroon. The latest, conducted in 2011, permitted the distribution of over eight million LLINs and increased substantially bed-net ownership across the country [38]. It is now estimated that over 60% of the population across the country own nets [1]. These initiatives and other factors such as the widespread use of pesticides in agriculture, particularly in urban agriculture, and the frequent use of repellent or insecticide spray might have contributed to the expansion of pyrethroid resistance across the country [19,20]. Although an important number of malaria outbreaks were reported across the country in 2013 [39], one could not confirm whether it was a consequence of the current levels of pyrethroid resistance in mosquito populations. Yet clinical and entomological studies conducted so far support no reduction in the intensity of malaria transmission in the cities of Yaoundé and Douala following implementation of control measures [40-42]. Because of the large number of confounding factors influencing malaria transmission, the real epidemiological impact of pyrethroid resistance on malaria transmission is still not easy to quantify, justifying the need for more operational studies [6,43].

Mosquitoes were found to be more susceptible to deltamethrin than to permethrin and this probably suggests different resistance mechanisms implicated. High *kdr* allele frequencies were recorded in vector populations of the two cities. Both *kdr* alleles L1014F and L1014S were detected, with, however, the L1014F highly prevalent. Although the two alleles were reported for the first time in Cameroon in 2006 [18], the L1014S allele frequency has not changed much since then. These findings suggest a probable limited role for L1014S allele in pyrethroid resistance in mosquito populations in Cameroon. Nevertheless the presence of L1014F and L1014S alleles in both *An. gambiae* and *An. coluzzii* is in agreement with the existence of introgression events between the two species [13,31].

Analysis of sequence variability of intron-1 voltage-gated sodium channel indicated a high variability of this sequence. Of the five haplotypes recorded, MS1 and MS3 were found to be the most prevalent. These haplotypes were found in previous studies [44]. MS1 was predominant in *An. coluzzii* samples originating from polluted and unpolluted sites and in *An. gambiae*, whereas MS3 was present only in *An. coluzzii* samples. The high prevalence of MS3 haplotype in *An. coluzzii* and its absence in *An. gambiae* contrasted with previous findings [44]. Moreover, *An. gambiae* and *An. coluzzii* samples from cultivated sites presented different haplotypes, supporting the very limited gene flow between the two species even in an area of sympatry and is consistent with earlier reports [45]. No evidence for a recent selective sweep on intron-1 sequence within samples originating from different breeding habitat was detected using Fu’s and Tajima Fs statistics. The highest negative value obtained with the Fu’s Fs statistic was recorded in unpolluted sites but the P-value was not significant. The diversity of intron-1 haplotypes in different types of habitats was consistent with the influence of different evolutionary forces (mutations, gene flow and selection). It is possible that the influence of evolutionary forces in shaping the genetic structure of mosquito populations might be counterbalanced by the ecological fitness of new genetic variants.

It is likely that the current evolution of pyrethroid resistance is maintained by both *kdr*-based and metabolic-based resistance mechanisms. Studies conducted so far suggested the implication of metabolic resistance in mosquitoes resistant to DDT, permethrin and deltamethrin in different parts of the country [36,37]. Several cytochrome P450 genes, such as CYP6P3, CYP6M2 and glutathion S transferase, were found to be over-expressed in mosquitoes resistant to DDT and pyrethroids [22]. Due to the large number of factors which could select for pyrethroid resistance [45] and the complexity of resistance mechanisms, it becomes important to take into consideration all these factors to design efficient vector control interventions.

## Conclusion

The present study supports rapid evolution of pyrethroid resistance prevalence in vector populations from the cities of Douala and Yaoundé. These changes need further attention in order to ensure the success of future control interventions. Although recent studies suggested the emergence of carbamate resistance [35] this compound alongside organophosphates could be used as alternative or associated to the use of pyrethroids for vector control. Other control strategies such as the use of larval source management might also be used as complement to treated nets to improve control of pyrethroid resistant mosquitoes particularly in urban settings.
